# Fermentation-Dependent Cell Wall-Associated Immunostimulation in a Red Pepper-Based *Lactiplantibacillus plantarum* CJLP243 Fermentate

**DOI:** 10.3390/foods15152736

**Published:** 2026-08-04

**Authors:** Sung-Hyun Jo, Na-Youn Hong, Bo-Gyeong Choi, Jiwon Chang, Suhyun Seo, Gayeong Lee, Hee-Yoon Ahn, Ji-Hyeon Song, Hee Taek Kim, Yung-Hun Yang, Jungoh Ahn, Hee-Kyoung Jung, Yun-Gon Kim

**Affiliations:** 1 Department of Chemical Engineering, Soongsil University, Seoul 06978, Republic of Korea; 2 CJ CheilJedang, Seoul 04560, Republic of Korea; 3 Department of Food Science and Technology, Chungnam National University, Daejeon 34134, Republic of Korea; 4 Department of Biological Engineering, College of Engineering, Konkuk University, Seoul 05029, Republic of Korea; 5 Biotechnology Process Engineering Center, Korea Research Institute of Bioscience and Biotechnology, Cheongju 28116, Republic of Korea

**Keywords:** *Lactiplantibacillus plantarum* CJLP243, red pepper-based fermentate, immunostimulation, cell wall-associated immunostimulation, pellet-derived lysate-insoluble fraction (PDIF), targeted metabolomics

## Abstract

Lactic acid bacteria-fermented food materials are promising sources of functional ingredients, but the active components underlying their bioactivity often remain unclear. We localized the macrophage-stimulatory activity of a red pepper-based fermentate prepared with *Lactiplantibacillus plantarum* CJLP243. Compared with the unfermented matrix and a non-fermented matrix supplemented with freeze-dried CJLP243, the fermentate induced greater IL-6 and TNF-α production in J774A.1 macrophages. Activity was reproducibly concentrated in the pellet-derived lysate-insoluble fraction (PDIF) and remained in residual debris after solvent extraction. LTA-directed fractionation and analytical screening did not support lipoteichoic acid as the principal determinant. Lysozyme markedly reduced activity, whereas proteinase K had a partial effect, a pattern consistent with a lysozyme-sensitive, cell wall-associated structure or cell wall–matrix complex, within which peptidoglycan-associated material remains a plausible candidate; contributions from co-associated components cannot be excluded. Targeted metabolomics revealed fermentation-associated changes in pellet-associated amino acid pools, consistent with broader adaptation of amino acid and nitrogen metabolism during fermentation; their biochemical linkage to cell-wall precursor pathways provides metabolic support rather than direct structural evidence. These findings identify a reproducible, process-linked insoluble fraction with macrophage-stimulatory activity and provide a framework for developing and standardizing fermentation-derived functional food ingredients.

## 1. Introduction

Fermentation-derived microbial preparations are being explored as alternatives to, or adjuncts to, live probiotics in applications where manufacturing consistency, product safety, and shelf stability are important [1,2,3,4]. The concept of postbiotics includes preparations of inanimate microorganisms and/or their components that confer a health benefit on the host, indicating that biological activity does not necessarily depend on viability [3]. This shift in focus is relevant to process development, because the active principle in a fermentation product may reside in non-viable cells, structural components, soluble metabolites, or combinations of these. At the same time, fermentates are complex systems composed of substrate-derived constituents, microbial structural materials, and molecules generated during fermentation. For that reason, it often remains unclear which component class is primarily responsible for the observed activity, and this uncertainty complicates process control, batch-to-batch reproducibility, and quality assessment [2,4]. The same issue affects downstream processing and potency testing, since different product formats may enrich different active fractions. From the standpoint of bioprocess development, identifying where the activity is localized is thus a necessary first step in defining reproducible functional materials.

In lactobacilli, immunomodulatory activity can arise not only from soluble metabolites but also from cell-envelope-associated components of Gram-positive bacteria [5,6]. Peptidoglycan (PGN), teichoic acids, surface proteins, and extracellular polysaccharides are all capable of interacting with the host innate immune system and influencing cytokine production, including IL-6 and TNF-α, in a strain- and context-dependent manner [5,6,7]. As general background, PGN-derived motifs are recognized by cytosolic pattern-recognition receptors such as NOD1 and NOD2, linking bacterial cell-wall structures to downstream innate immune signaling [6,7]. In this setting, recognition of bacterial cell wall signals should not be interpreted simply as nonspecific inflammatory toxicity; rather, controlled sensing of such structures may contribute to innate immune priming and host defense [8,9]. Despite this, many studies in the probiotic field still use whole cells or crude lysates as test materials. That approach is useful for initial screening, but it makes it difficult to distinguish metabolite-driven effects from activity associated with insoluble structural fractions.

Fermentation conditions can influence both the composition of the cell envelope and the extent to which structural determinants are exposed to host sensing [10,11]. Environmental variables such as pH, temperature, salt, and stress can alter surface properties, cell-envelope homeostasis, and envelope remodeling in lactic acid bacteria [10,11,12,13]. These changes are not only relevant to adhesion and environmental survival, but also to the amount and accessibility of immunologically active material retained in insoluble fractions or released after enzymatic perturbation. Work on *Lactobacillus plantarum* OLL2712 further showed that culture conditions can affect immunomodulatory activity, supporting the broader view that process conditions can shape functionality even within a single strain [14]. This point is particularly important for fermentation-derived products, because the active material may not simply be “present” or “absent”; rather, fermentation can shift where that material is found and how readily it is detected in fractionated preparations. Because fermentates combine substrate-derived compounds, microbial metabolites, inactivated cells, and cell-envelope structures, whereas whole-cell or crude-lysate screening cannot resolve which component class is active, a fraction-resolved strategy combined with orthogonal enzyme-sensitivity tests is needed to localize the activity and narrow its chemical basis—an essential first step toward developing process-linked, reproducible functional materials.

On this basis, we investigated a red pepper-based fermentation system inoculated with *Lactiplantibacillus plantarum* CJLP243 to determine how fermentation defines the localization and structural characteristics of macrophage-stimulatory activity within a complex food matrix. As a primary comparison, we evaluated the unfermented substrate, the CJLP243 fermentate, and a non-fermented matrix supplemented with freeze-dried CJLP243 to distinguish fermentation-dependent activity from effects attributable to bacterial cell addition alone. We then used fraction-resolved cytokine assays to localize the activity among supernatant-, pellet-, and subfractionated materials, and combined this workflow with LTA-directed fractionation and analysis, enzymatic dissection, and targeted LC–MS/MS-based metabolomics to narrow the likely structural basis of the active material. The aim of this work was to localize the immunostimulatory activity within the fermentate and to narrow its likely chemical basis using orthogonal functional and analytical tests, rather than to achieve complete molecular identification of the active species. The novelty of this study lies not in simply demonstrating cytokine induction by a lactic acid bacterium or its fermentate, but in resolving a reproducible, process-dependent, lysozyme-sensitive insoluble fraction associated with CJLP243-mediated macrophage stimulation in a red pepper-based food system. Thus, the aim of this work was not to claim direct therapeutic efficacy, but to define a fermentation-linked immunostimulatory property and provide a process-relevant framework for quality-oriented development of functional fermented food ingredients.

## 2. Materials and Methods

### 2.1. Bacterial Strains and Preparation of the Red Pepper-Based Substrate System

A red pepper-based fermentation system was prepared using freeze-dried *Lactiplantibacillus plantarum* CJLP243 (KCCM11045P). The substrate mixture consisted of 3.0 wt% crystalline fructose, 2.0 wt% red pepper powder, 1.5 wt% minced ginger, 0.5 wt% sodium citrate, and 0.2 wt% anhydrous citric acid in water. The mixture was sterilized and then cooled to 37 °C. After cooling, the freeze-dried CJLP243 powder was inoculated at 0.0167 wt%, and the mixture was fermented at 37 °C for 24 h to obtain the final fermented broth. Throughout this study, the unfermented raw material mixture was designated as “Keybase”, and the corresponding fermented product was designated as “CJLP243 Fermentate.” Unless otherwise stated, all chemicals and reagents were purchased from Sigma-Aldrich (St. Louis, MO, USA).

### 2.2. Preparation of Fermented and Comparison Samples

To examine fermentation-dependent and strain-dependent differences, the following comparison groups were prepared and used in this study: Keybase, CJLP243 Fermentate, CJLP243 FD add, and LP STD Fermentate. Keybase was defined as the raw material base without inoculation or fermentation. CJLP243 Fermentate referred to the raw material mixture fermented with freeze-dried CJLP243. CJLP243 FD add referred to the non-fermented raw material mixture supplemented with freeze-dried CJLP243. LP STD Fermentate referred to raw materials fermented with the reference strain *L. plantarum* KCTC 13093.

### 2.3. Fractionation of Fermentates and Keybase Samples

For sample preparation, Keybase and CJLP243 Fermentate were first centrifuged at 14,000 rpm for 10 min to separate the supernatant and pellet fractions. The supernatant was further divided into high molecular weight (>10 kDa) and low molecular weight (<10 kDa) fractions using a 10 kDa molecular weight cut-off filter. The low molecular weight fraction was subsequently subjected to liquid–liquid extraction with chloroform/methanol mixture to further separate metabolites according to polarity. For chloroform/methanol extraction, samples were mixed with chloroform and methanol to a final ratio of chloroform:methanol:water = 1:2:0.8, vortexed for 1 min, and incubated at room temperature for 10 min [15]. Additional chloroform and deionized water were then added to adjust the final ratio to 2:2:1.8, and the mixture was centrifuged at 4000 rpm for 5 min. The separated organic and aqueous layers were collected. The organic fraction was dried under a nitrogen stream, and the aqueous fraction was dried using a SpeedVac (Gimpo, Republic of Korea). For pellet fractionation, the cell pellet obtained from CJLP243 Fermentate was disrupted by ultrasonication (35% amplitude, 5 s pulse, 5 s rest, 10 min) on ice to generate a cell lysate, which was then centrifuged again at 14,000 rpm for 10 min to obtain intracellular soluble and insoluble fractions. The pellet-derived lysate-insoluble fraction is termed the PDIF. Dried fractions were reconstituted before use as follows. The aqueous fraction was resuspended in PBS to its original volume. The organic fraction was first dissolved in DMSO at one-tenth of the original volume and then diluted with PBS to the original volume. The PDIF was resuspended in PBS to the original volume and homogenized by ultrasonication on ice (35% amplitude; 5 s pulse, 5 s rest; 10 min total) before use.

### 2.4. Cell Culture and Cytokine Assays

The murine macrophage cell line J774A.1 was obtained from the Korean Cell Line Bank (KCLB). Cells were maintained in Dulbecco’s modified Eagle’s medium (DMEM; Gibco, Grand Island, NY, USA) supplemented with 10% heat-inactivated fetal bovine serum (Gibco, Grand Island, NY, USA), 1% L-glutamine (Gibco, Grand Island, NY, USA), 100 IU/mL penicillin G, and 100 µg/mL streptomycin (Gibco, Grand Island, NY, USA) at 37 °C in a humidified incubator containing 5% CO_2_. For cytokine assays, J774A.1 cells were seeded into 96-well plates at a density of 5 × 10^4^ cells/well in 150 μL of culture medium and incubated for 3 h to allow monolayer formation. Immediately before treatment, each sample was diluted 10- or 20-fold in culture medium. The cells were then treated with 100 μL of each prepared sample or fraction for 24 h. After incubation, the culture medium was centrifuged at 1400 rpm for 10 min, and the supernatant was collected.

TNF-α and IL-6 concentrations in the culture supernatants were quantified using Mouse DuoSet ELISA kits (R&D Systems, Minneapolis, MN, USA; TNF-α, DY406-05; IL-6, DY410-05) according to the manufacturer’s instructions. Briefly, capture antibodies were coated onto ELISA plates for 24 h at room temperature. After incubation with samples or standards for 2 h, biotinylated detection antibodies were applied for an additional 2 h. A colorimetric reaction was then developed using streptavidin-HRP and TMB substrate and terminated with 2 N H_2_SO_4_. Absorbance was measured at 450 nm with a correction wavelength of 540 nm using a spectrophotometer (BioTek, Winooski, VT, USA). LPS (10 μg/mL, Sigma-Aldrich, St. Louis, MO, USA) and untreated cells were used as positive and negative controls, respectively.

### 2.5. Lipoteichoic Acid (LTA)-Targeted Fractionation and Analysis

To examine the potential contribution of LTA to the immunostimulatory activity, the pellet of CJLP243 Fermentate was subjected to n-butanol extraction followed by hydrophobic interaction chromatography, as described previously with minor modifications [16,17]. Briefly, 50 mL of CJLP243 Fermentate was centrifuged at 4000 rpm for 10 min, and the supernatant was discarded. The cell pellet was washed twice with 5 mL of resuspension buffer (50 mM sodium citrate, pH 4.7) and finally resuspended in 5 mL of the same buffer. Cells were disrupted by ultrasonication (35% amplitude, 5 s pulse, 5 s rest, 10 min) on an ice block. An equal volume (5 mL) of n-butanol was added, and the mixture was incubated for 30 min at room temperature with continuous mixing. After centrifugation at 4000 rpm for 30 min, the n-butanol layer and aqueous layer were collected separately. The n-butanol-soluble fraction was dried under a nitrogen stream, reconstituted in DMSO at one-tenth of the original volume, and diluted with PBS to the original volume. The aqueous fraction was dialyzed against pyrogen-free water for 48 h, dried using a SpeedVac concentrator, and reconstituted in PBS. Each fraction was then used in cell-based cytokine assays.

For LTA isolation, the aqueous fraction obtained from n-butanol extraction was sequentially dialyzed, first against pyrogen-free water overnight and then against 0.1 M sodium citrate buffer containing 15% n-propanol (pH 4.7) overnight. Hydrophobic interaction chromatography was performed using octyl-Sepharose CL-4B (Sigma-Aldrich, St. Louis, MO, USA), and putative LTA-containing fractions were eluted using 0.1 M sodium citrate buffer containing 35% and 45% n-propanol.

The presence of LTA in the recovered fractions was assessed by matrix-assisted laser desorption/ionization time-of-flight mass spectrometry (MALDI–TOF MS). All eluted fractions were pooled, dialyzed against pyrogen-free water for 48 h, and dried using a SpeedVac. The dried material was subjected to mild acid hydrolysis to release the glycolipid anchor [18]. The dried sample was dissolved in 98% (*v*/*v*) aqueous acetic acid by sonication for 30 min and then heated at 100 °C for 3 h. After vacuum evaporation of the solvent, the sample was partitioned by extraction with chloroform/methanol/water (1:1:0.9, *v*/*v*/*v*), and the glycolipid-containing organic layer was collected and dried. The dried glycolipid fraction was reconstituted in MS grade water, 1 µL of sample was co-spotted with 1 µL of 30 mg/mL 2,5-dihydroxybenzoic acid (DHB; in 70% acetonitrile/water) onto an MSP 96 target polished steel MALDI plate and air-dried at room temperature. Analyses were performed on a Bruker Daltonics Microflex LRF MALDI–TOF MS system using FlexControl 3.0 software.

LTA was further estimated quantitatively using an inorganic phosphate assay [19]. Phosphate standards were prepared at concentrations ranging from 4 mM to 0.25 mM. Standards and chromatography fractions (100 µL each) were transferred into 2 mL microtubes in a fume hood, followed by addition of 200 µL of ashing solution (2 M H_2_SO_4_, 0.44 M HClO_4_). The tubes were incubated at 120 °C for 3 h on a heat block. After cooling, 1 mL of reducing solution (3 mM ammonium molybdate, 0.25 M sodium acetate, 1% ascorbic acid) was added. Samples were incubated at 45 °C for 2 h with occasional inversion. A 250 µL aliquot was transferred to a 96-well plate, and absorbance was measured at 700 nm using a UV–vis spectrometer.

### 2.6. Enzymatic Dissection of the Active PDIF

For component analysis of the PDIF, samples from Keybase, CJLP243 Fermentate, and the LP STD Fermentate were subjected to enzymatic or chemical treatment targeting distinct cell-envelope components. For lysozyme treatment, PDIF samples were resuspended in 3 mL of lysozyme (5 mg/mL in PBS) and incubated at 37 °C for 2 h. For proteinase K treatment, samples were resuspended in 3 mL of proteinase K (100 µg/mL in 100 mM Tris–HCl, pH 8.0) and incubated at 60 °C for 2 h. After incubation, both treatments were centrifuged at 4000 rpm for 10 min, washed once with PBS, re-centrifuged under the same conditions, and finally resuspended in PBS to the original sample volume. For hydrofluoric acid (HF) treatment, PDIF samples were resuspended in 0.5 mL of 48% HF and incubated at 4 °C for 24 h. Samples were dried under a nitrogen stream, reconstituted in deionized water, dried again using a SpeedVac, and finally resuspended in PBS to the original sample volume.

### 2.7. Targeted LC–MS/MS-Based Metabolomics

Targeted metabolomic profiling was performed as described in our previous studies [20,21]. Briefly, cell pellets recovered from 10 mL of each preparation (Keybase, CJLP243 Fermentate, and CJLP243 FD add) were extracted with 1 mL of pre-chilled 80% methanol (−80 °C) and incubated at −80 °C for 4 h. After centrifugation at 4000 rpm for 10 min at 4 °C, the metabolite-containing supernatant was collected. The residual pellet was re-extracted with a further 1 mL of 80% methanol (−80 °C) for 1 h, and the two supernatants were pooled. The pooled extracts were dried using a SpeedVac concentrator and reconstituted in 0.1 mL of HPLC-grade water.

Targeted analysis was carried out on a 1260 Infinity Binary LC coupled to a 6420 Triple Quadrupole mass spectrometer (Agilent, Santa Clara, CA, USA). Each metabolite sample was injected onto an XBridge Amide column (4.6 × 250 mm, 3.5 µm particle size; Waters, Milford, MA, USA). Mobile phase A consisted of water/acetonitrile (95:5) containing 20 mM ammonium acetate and 20 mM ammonium hydroxide, and mobile phase B consisted of 100% acetonitrile. The chromatographic gradient was programmed as follows: 85% B at 0 min, 42% B at 5 min, 0% B at 16 min, 0% B at 24 min, 85% B at 25 min, and 85% B at 32 min, at a flow rate of 0.4 mL/min. The capillary temperature was maintained at 300 °C, and the electrospray ionization voltage was set to 4 kV. Primary metabolites were separated and quantified using an in-house multiple reaction monitoring (MRM) library established for primary metabolic intermediates, and MS peak areas were extracted using Agilent MassHunter Qualitative Analysis software (version B.07.00). Statistical analysis of the normalized peak-area data was performed using MetaboAnalyst 5.0 [22]. Statistical significance was assessed using false discovery rate (FDR)-adjusted *p*-values, and metabolites showing a fold-change ≥ 2 with an FDR-adjusted *p*-value < 0.05 were defined as quantitatively significant.

### 2.8. Statistical Analysis

Unless otherwise stated, biological replicates (*n* = 3 for cytokine assays; *n* = 4 for metabolomics) represent independently repeated fraction-preparation and cell-treatment experiments rather than independent fermentation batches. In the batch-reproducibility experiment, however, the three biological replicates were three independently prepared fermentation batches. For each biological replicate, ELISA measurements were performed in duplicate, and the mean of the two technical replicates was used as the biological-replicate value. Data are presented as the mean ± SD of the biological replicates. Statistical analyses were performed using GraphPad Prism (https://www.graphpad.com/, GraphPad Software, Boston, MA, USA). Normality and homogeneity of variance were evaluated using the Shapiro–Wilk and Brown–Forsythe tests, respectively, before parametric analysis. After these assumptions were confirmed, group differences were evaluated by one- or two-way ANOVA followed by Tukey’s multiple-comparisons test, as appropriate. Significance is denoted as * *p* < 0.05, ** *p* < 0.01, *** *p* < 0.001; ns, not significant.

## 3. Results and Discussion

### 3.1. Red Pepper-Based CJLP243 Fermentation System and Fermentation-Dependent Immunostimulatory Activity

**Figure 1:**
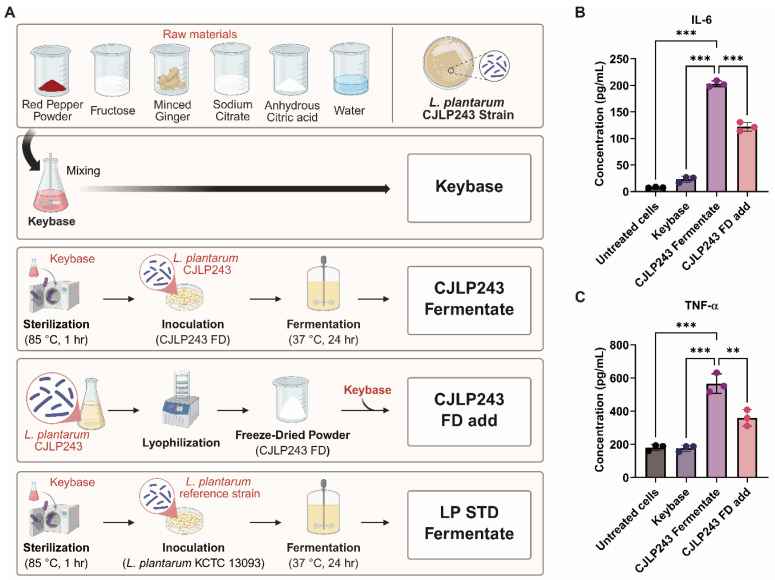
Establishment of a red pepper-based *Lactiplantibacillus plantarum* CJLP243 fermentation system and fermentation-dependent immunostimulatory activity. (**A**) Schematic overview of the red pepper-based fermentation system used in this study. Red pepper powder, or a red pepper-containing mixture, was inoculated with *L. plantarum* CJLP243 and incubated under controlled fermentation conditions to generate the test fermentate. The major comparison groups used throughout the study are also defined, including the unfermented substrate (Keybase), fermented controls, CJLP243 Fermentate, non-fermented CJLP243-added control (CJLP243 FD add), and reference strain *L. plantarum* fermentate (LP STD Fermentate). This illustration was created using BioRender.com. (**B**) IL-6 production and (**C**) TNF-α production in J774A.1 cells treated with the indicated samples. The CJLP243 Fermentate produced substantially stronger cytokine responses than the CJLP243 FD add or Keybase, indicating that fermentation was required to generate or expose the immunostimulatory activity examined in the subsequent fractionation analyses. Statistical significance was assessed by one-way ANOVA. Significance is denoted as ** *p* < 0.01, *** *p* < 0.001.

This study was initiated using a red pepper-based fermentation system inoculated with *L. plantarum* CJLP243, a kimchi-derived lactic acid bacterium. Red pepper powder was used as a food matrix because it is a major ingredient in kimchi-type vegetable fermentations and contains plant-derived bioactive compounds that may be modified during microbial fermentation. Previous studies have also indicated that red pepper-containing matrices can influence microbial succession during kimchi fermentation and that *L. plantarum* fermentation can alter the functional properties of spice-derived suspensions [23,24]. Therefore, the present system was designed to examine whether fermentation with CJLP243 could generate an immunologically active fraction in a red pepper-based food matrix, rather than simply evaluating the activity of bacterial biomass added to the substrate (Figure 1A). To distinguish fermentation-dependent effects from those caused by strain addition alone, comparison groups were prepared as Keybase, CJLP243 Fermentate, and CJLP243 FD add (Figure 1A). This design was necessary because fermented food materials may contain multiple immunologically relevant components, including substrate-derived compounds, microbial metabolites, inactivated microbial cells, and bacterial cell-envelope structures.

Initial screening showed that the CJLP243 Fermentate induced higher IL-6 and TNF-α production in J774A.1 macrophages than the Keybase and CJLP243 FD add (Figure 1B,C). In contrast, simple addition of freeze-dried CJLP243 to the red pepper-based substrate did not reproduce the cytokine-inducing activity observed after fermentation (Figure 1B,C). These results indicate that the immunostimulatory property of the CJLP243 system was not explained solely by the presence of bacterial biomass. Rather, the activity appeared to be associated with a fermentation-dependent change in the red pepper-based matrix. This finding provided the basis for subsequent experiments aimed at localizing the active fraction and defining its likely structural characteristics.

### 3.2. Pellet-Derived Lysate-Insoluble Fraction as the Major Immunostimulatory Fraction

**Figure 2:**
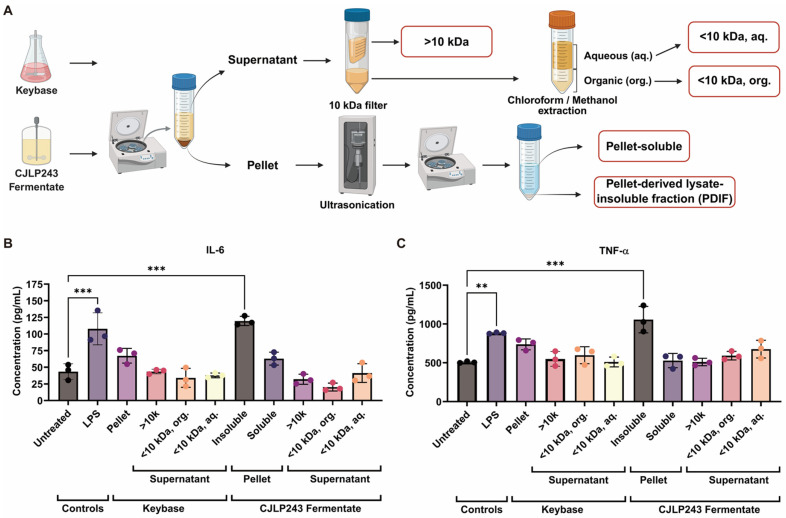
Fraction-resolved localization of immunostimulatory activity to the PDIF. (**A**) Workflow used to fractionate Keybase and CJLP243 fermentate into supernatant and pellet fractions, followed by secondary separation into high- and low-molecular-weight (>10 kDa and <10 kDa) supernatant fractions and pellet lysate-soluble and lysate-insoluble fractions (PDIF). The <10 kDa supernatant was further partitioned by methanol/chloroform extraction into aqueous (aq.) and organic (org., chloroform) phases. This illustration was created using BioRender.com. (**B**) IL-6 production and (**C**) TNF-α production in J774A.1 cells treated with the indicated fractions. Supernatant-derived fractions showed little or no cytokine-inducing activity, whereas the PDIF of the CJLP243 Fermentate induced the strongest responses. These results indicate that the major immunostimulatory determinant of the fermentate is concentrated in the PDIF rather than in soluble metabolite pools. Statistical significance was assessed by one-way ANOVA. Significance is denoted as ** *p* < 0.01, *** *p* < 0.001.

To localize the active component, the Keybase and CJLP243 Fermentate were fractionated into supernatant- and pellet-derived fractions. The supernatant was further separated into high- and low-molecular-weight fractions, whereas the pellet was separated into lysate-soluble and lysate-insoluble fractions; hereafter, the pellet-derived lysate-insoluble fraction is termed the PDIF (Figure 2A). Most supernatant-derived fractions showed little or no induction of IL-6 or TNF-α, and the pellet lysate-soluble fraction also induced only limited cytokine production (Figure 2B,C). In contrast, the PDIF from CJLP243 Fermentate produced the strongest cytokine responses among all fractions tested (Figure 2B,C). The fractionation results indicate that the major immunostimulatory activity was preferentially recovered in the PDIF rather than in soluble metabolite pools. This observation shifted the mechanistic focus from low-molecular-weight fermentation products to cell-associated or matrix-associated structures. In lactic acid bacteria, the Gram-positive cell envelope contains peptidoglycan, teichoic acids, cell-wall polysaccharides, surface proteins, and other associated macromolecules that may participate in host–microbe recognition [12,25]. Therefore, the enrichment of activity in the PDIF raised the possibility that the active material was related to bacterial cell-envelope components generated, exposed, or stabilized during CJLP243 fermentation.

At the same time, the activity should not be interpreted as arising from bacterial material alone, because the fraction was obtained from a red pepper-based fermented food matrix. Accordingly, the data are consistent with a food-bioprocessing model in which CJLP243 fermentation generates a matrix-associated particulate fraction with macrophage-stimulatory activity. We hypothesize that insoluble plant-derived particles, bacterial cell-wall fragments, and cell–matrix complexes may jointly influence the physicochemical state of this fraction (the PDIF). Because these components were not individually isolated or characterized, however, this model and the respective contributions of its components require direct experimental testing.

### 3.3. Batch-to-Batch Reproducibility of the Active PDIF

**Figure 3:**
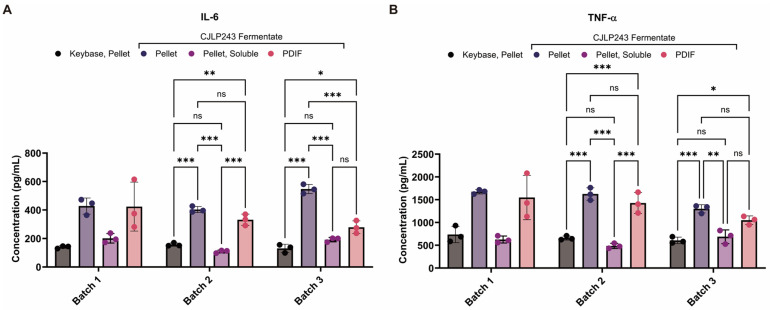
Batch-to-batch reproducibility of the active PDIF from CJLP243 fermentate. (**A**) IL-6 production and (**B**) TNF-α production induced by pellet-derived soluble fraction and PDIF prepared from independent batches of Keybase and CJLP243 Fermentate. The pellet-derived fractions of CJLP243 Fermentate reproducibly showed elevated cytokine-inducing activity across independent batches, with activity consistently retained in the PDIF. Statistical significance was assessed by two-way ANOVA. Significance is denoted as * *p* < 0.05, ** *p* < 0.01, *** *p* < 0.001; ns, not significant.

To determine whether the active fraction was reproducibly generated, three independent production batches were prepared and evaluated using the same fractionation and cytokine assay workflow. Across all three independent batches, CJLP243 Fermentate reproducibly showed elevated cytokine-inducing activity in the pellet-derived fractions, and substantial activity was consistently retained in the PDIF (Figure 3A,B). The repeated recovery of activity in the same fraction suggests that the immunostimulatory property was not batch-specific. This point is relevant for the development of fermented food-derived functional materials, because biological activity must be linked to a reproducible process attribute rather than to a single preparation. The consistent enrichment of activity in the PDIF also indicates that this fraction may serve as a practical target for further structural characterization and future process optimization. The reproducibility of activity localization also narrowed the interpretation of the system. If the response had been driven mainly by unstable soluble metabolites, marked differences in the supernatant-derived fractions might have been expected among production batches. Instead, the repeated enrichment of activity in the PDIF suggests that CJLP243 fermentation repeatedly generated, exposed, or stabilized a particulate immunostimulatory material. Based on this result, subsequent analyses focused on the physicochemical properties of the PDIF.

### 3.4. Retention of Immunostimulatory Activity in the Non-Extractable Insoluble Residue and Limited Involvement of Lipoteichoic Acid

**Figure 4:**
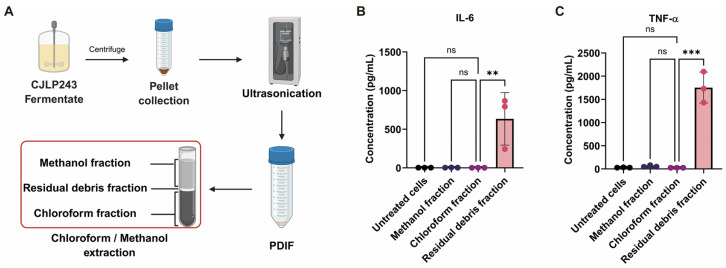
Subfractionation of the active PDIF by chloroform/methanol extraction. (**A**) Scheme for subfractionation of the PDIF into methanol-soluble and chloroform-soluble, and residual debris fractions. This illustration was created using BioRender.com. (**B**) IL-6 production and (**C**) TNF-α production in J774A.1 cells treated with the indicated subfractions. Cytokine-inducing activity was retained predominantly in the residual debris fraction, whereas the solvent-extractable fractions showed little or no activity. These findings indicate that the principal active material is associated with a non-extractable insoluble component. Statistical significance was assessed by one-way ANOVA. Significance is denoted as ** *p* < 0.01, *** *p* < 0.001; ns, not significant.

Because the activity was enriched in the PDIF, chloroform/methanol extraction was used to examine whether solvent-extractable components contributed to the response. The PDIF was separated into methanol, chloroform, and residual debris fractions (Figure 4A). The methanol and chloroform fractions induced no IL-6 and TNF-α production, whereas cytokine-inducing activity was retained mainly in the residual debris fraction (Figure 4B,C). These results suggest that the major active material was not readily recovered as a solvent-extractable component. Instead, the activity remained associated with a non-extractable insoluble residue. This behavior is more consistent with a structurally stable cell wall- or matrix-associated material than with a freely diffusible metabolite or solvent-extractable amphipathic compound. This finding also provided a rationale for examining LTA, a representative amphipathic cell-envelope component of Gram-positive bacteria. Because LTA has been implicated in immune responses induced by lactic acid bacteria, it was necessary to determine whether LTA could account for the cytokine-inducing activity retained in the PDIF [26].

**Figure 5:**
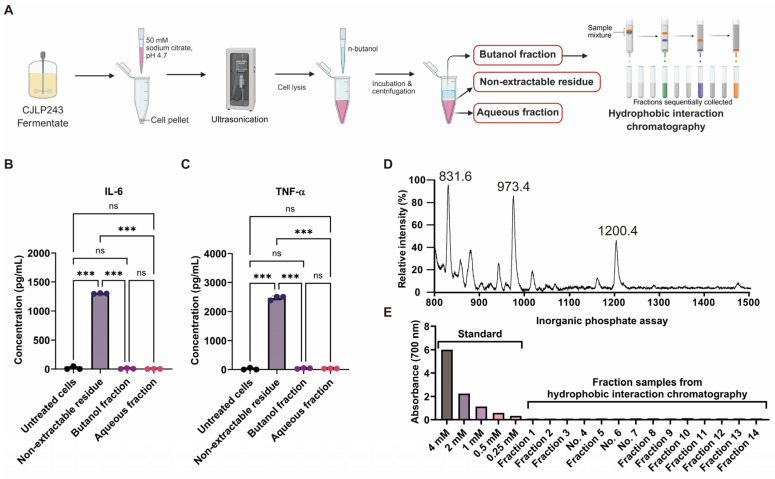
LTA-directed extraction and analytical characterization do not support LTA as the major immunostimulatory determinant. (**A**) Workflow for n-butanol extraction and hydrophobic interaction chromatography used to isolate putative lipoteichoic acid (LTA)-containing fractions from the cell pellet of CJLP243 Fermentate. This illustration was created using BioRender.com. (**B**) IL-6 production and (**C**) TNF-α production in J774A.1 cells treated with the indicated fractions obtained during LTA-directed fractionation. Statistical significance was assessed by one-way ANOVA. (**D**) MALDI-TOF MS profile of the glycolipid anchor obtained after pooling all HIC elution fractions, followed by acetic acid hydrolysis and chloroform/methanol/water partitioning. (**E**) Inorganic phosphate measurements of fraction eluted using 0.1 M sodium citrate buffer containing 35% and 45% n-propanol. Both the n-butanol partitioned fractions tested in cell-based assays (**B**,**C**) and the chromatographically enriched fractions analyzed by MS and phosphate quantification (**D**,**E**) failed to provide evidence supporting LTA as a principal active component. Together with the subfractionation results shown in Figure 4, these data argue against LTA as the principal active determinant in the CJLP243 Fermentate. Significance is denoted as *** *p* < 0.001; ns, not significant.

To evaluate whether LTA could account for the activity retained in the PDIF, the pellet from CJLP243 Fermentate was subjected to n-butanol extraction and hydrophobic interaction chromatography, a workflow used for LTA-directed enrichment (Figure 5A). Because LTA from lactic acid bacteria is a structurally heterogeneous amphiphilic polymer containing glycerolphosphate repeats, variable substituents, and glycolipid anchor structures, MALDI–TOF MS was interpreted as a qualitative screening tool rather than as definitive structural identification. Fractions obtained by LTA-directed enrichment showed little or no cytokine-inducing activity in J774A.1 cells (Figure 5B,C). In addition, MALDI–TOF MS profiling did not yield an interpretable LTA-like ion pattern suitable for structural assignment, and inorganic phosphate analysis did not show substantial enrichment of phosphate-containing material linked to cytokine-inducing activity (Figure 5D,E). Taken together, the functional and analytical results do not support LTA as the principal immunostimulatory determinant under the conditions tested. Nevertheless, a minor contribution from low-abundance or structurally heterogeneous LTA cannot be excluded by the enrichment and screening methods used here. These findings redirected the subsequent analysis toward non-extractable, lysozyme-sensitive cell-wall material, and enzymatic and chemical sensitivity analyses were therefore performed to further characterize the active PDIF.

### 3.5. Lysozyme-Sensitive Cell Wall-Associated Activity in the PDIF

**Figure 6:**
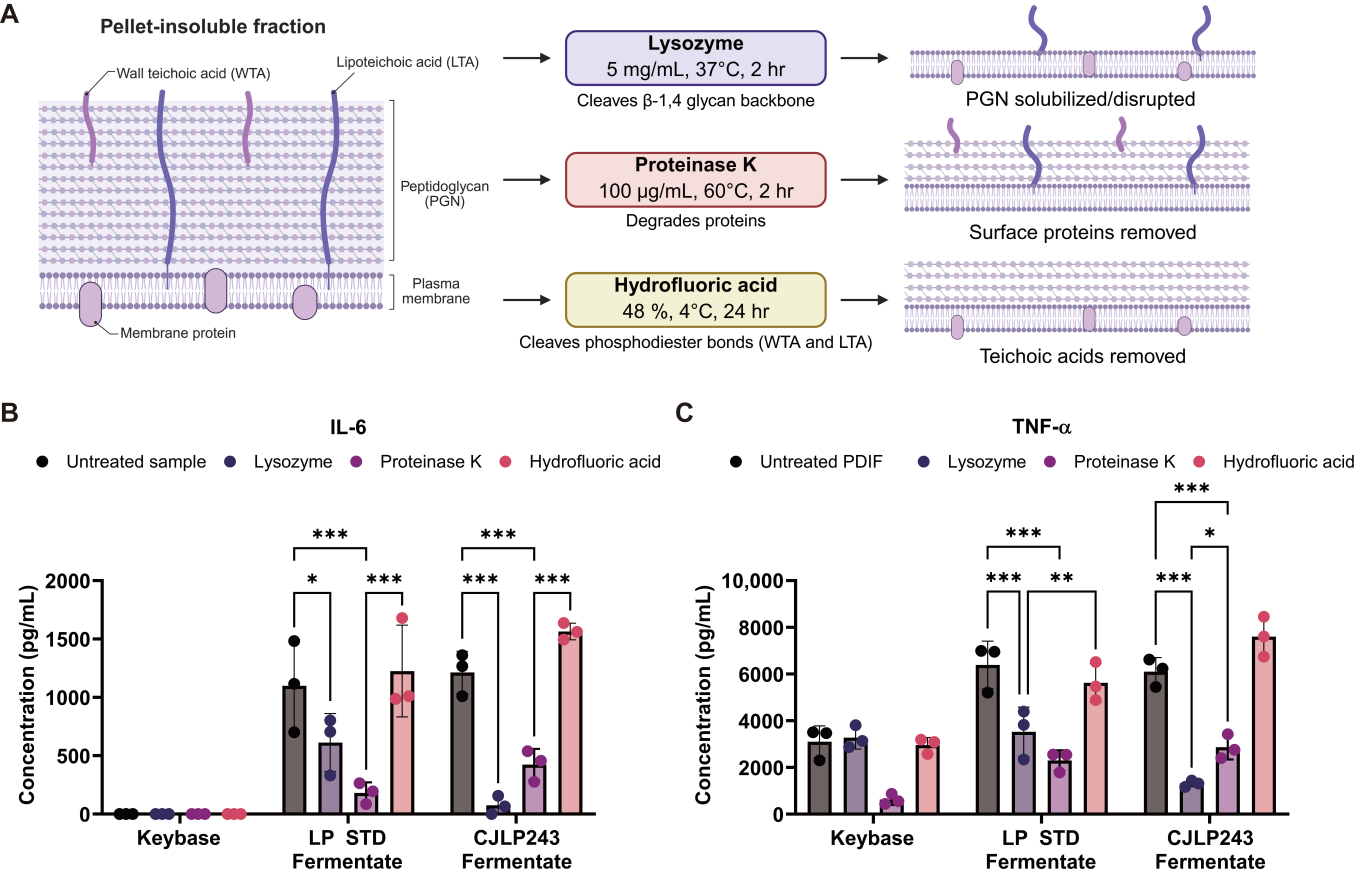
Enzymatic dissection of the active PDIF supports association with a lysozyme-sensitive cell wall-associated determinant. (**A**) Schematic representation of the enzymatic and chemical treatments applied to the PDIF. Lysozyme was used to target the glycan backbone of peptidoglycan, proteinase K to degrade proteinaceous components, and hydrofluoric acid to remove lipoteichoic acid-related structures. This illustration was created using BioRender.com. (**B**) IL-6 production and (**C**) TNF-α production after treatment of PDIFs from Keybase, CJLP243 Fermentate, and the LP STD Fermentate. The CJLP243 Fermentate showed marked loss of activity after lysozyme treatment, partial reduction after proteinase K treatment, and no reduction after hydrofluoric acid treatment. This pattern is consistent with a dominant contribution of a lysozyme-sensitive cell wall-associated determinant. Statistical significance was assessed by two-way ANOVA. Significance is denoted as * *p* < 0.05, ** *p* < 0.01, *** *p* < 0.001.

To further define the structural basis of the active fraction, PDIF was treated with lysozyme, proteinase K, or hydrofluoric acid (HF) (Figure 6A). In the PDIF from CJLP243 Fermentate, lysozyme treatment markedly reduced both IL-6 and TNF-α induction, whereas proteinase K caused only partial reduction. By contrast, hydrofluoric acid treatment did not reduce cytokine-inducing activity, indicating that the dominant activity was lysozyme-sensitive but not hydrofluoric acid-labile (Figure 6B,C). Notably, the PDIF from Keybase and the LP STD Fermentate showed a different sensitivity pattern, with proteinase K exerting a relatively greater effect than lysozyme (Figure 6B,C). Lysozyme cleaves the β-1,4 linkage between N-acetylmuramic acid and N-acetylglucosamine in the peptidoglycan glycan backbone. Therefore, the marked reduction in cytokine-inducing activity recovered from the washed, post-treatment insoluble fraction indicates that retention of activity in the PDIF depends substantially on the integrity of a lysozyme-sensitive cell-wall-associated framework; it does not identify peptidoglycan as the sole active ligand. On this basis, the enzyme-sensitivity profile is consistent with the involvement of a lysozyme-sensitive, cell wall-associated structure in the PDIF of CJLP243 Fermentate. However, because lysozyme cleavage of the β-1,4 glycan backbone can also release or destabilize macromolecules that are physically associated with peptidoglycan, including surface- or matrix-bound proteins, wall polysaccharides, teichoic acids, and peptidoglycan–plant-fiber complexes, these data localize the activity to a lysozyme-releasable, cell-wall-associated compartment but do not, by themselves, establish peptidoglycan as the sole active molecule. This interpretation is consistent with previous studies showing that lactic acid bacterial peptidoglycan and peptidoglycan-related structural variation can influence macrophage cytokine responses [27,28].

The partial reduction observed after proteinase K treatment may reflect the contribution of proteinaceous components to the integrity, exposure, or accessibility of the active structure, rather than indicating that proteins are the primary active component. The absence of a reduction after HF treatment indicates that the active determinant is unlikely to be a phosphate-rich, hydrofluoric acid-labile structure. HF cleaves phosphodiester bonds in both wall teichoic acid and lipoteichoic acid. The unchanged activity after HF treatment therefore suggests that HF-sensitive teichoic acid structures are unlikely to be the principal contributors to the observed activity. However, a minor contribution cannot be ruled out because HF digestion may have been incomplete or the insoluble particulate matrix may have restricted HF access to these structures.

The different sensitivity patterns observed among CJLP243 Fermentate, Keybase, and the LP STD Fermentate further suggest that not all *L. plantarum*-related fermentates rely on the same structural basis for macrophage activation. In the CJLP243 system, fermentation appears to produce or expose a lysozyme-sensitive, cell wall-associated active material.

### 3.6. Fermentation-Dependent Generation or Exposure of a CJLP243-Specific Active Structure

**Figure 7:**
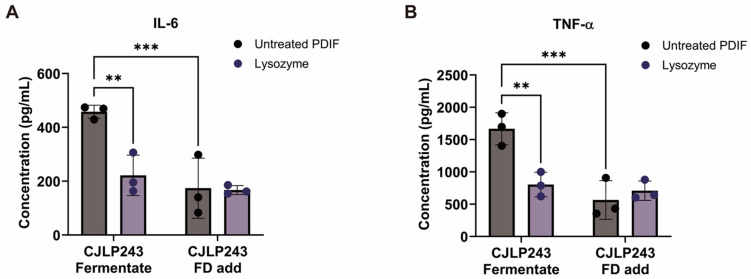
Fermentation is required for the acquisition of the CJLP243-specific lysozyme-sensitive immunostimulatory determinant. (**A**) IL-6 production and (**B**) TNF-α production in J774A.1 cells treated with PDIFs from CJLP243 Fermentate and CJLP243 FD add, with or without lysozyme treatment. The CJLP243 FD add group showed little cytokine-inducing activity in the PDIF, whereas the fermented CJLP243 sample retained strong activity and a characteristic lysozyme-sensitive response pattern. These findings indicate that fermentation is required to generate or expose the CJLP243-specific active determinant. Statistical significance was assessed by two-way ANOVA. Significance is denoted as ** *p* < 0.01, *** *p* < 0.001.

To distinguish the effect of fermentation from that of strain addition alone, the PDIFs from CJLP243 Fermentate and CJLP243 FD add were compared, with and without lysozyme treatment. The CJLP243 FD add fraction showed markedly lower IL-6 and TNF-α induction than the corresponding fraction from CJLP243 Fermentate (Figure 7A,B). Moreover, lysozyme treatment significantly reduced cytokine induction in the CJLP243 Fermentate, whereas it had no effect on the already low activity of the CJLP243 FD add fraction (Figure 7A,B). These results indicate that the immunostimulatory activity was not simply due to the presence of CJLP243 biomass in the substrate, and that the lysozyme-sensitive active determinant was specifically acquired through fermentation.

Previous studies have shown that non-viable or heat-treated *L. plantarum* cells can stimulate macrophage cytokine production through innate immune signaling pathways [29]. The present results extend this concept by showing that, in a red pepper-based matrix, fermentation was required to generate or expose the CJLP243-associated structure responsible for the strongest activity. Fermentation may alter the bacterial cell envelope, promote partial autolysis, increase the accessibility of peptidoglycan-associated motifs, or stabilize cell-wall material through interaction with insoluble food matrix components. Although the exact molecular architecture remains to be resolved, the data support a process-dependent structural transition rather than a constitutive property of the strain alone.

We cannot exclude the possibility that nutrient depletion and organic-acid stress during the 24 h fermentation promoted partial autolysis, releasing intracellular components (e.g., genomic DNA, RNA, or cytoplasmic proteins) that physically adsorbed onto the insoluble red pepper matrix. Such autolysis and cell-wall turnover may be components of the fermentation-dependent process and could contribute to the generation, exposure, or matrix association of the active determinant. The marked lysozyme effect, together with the partial proteinase K effect, remains consistent with an important contribution from a cell-wall scaffold and associated proteinaceous components. However, these findings do not exclude intracellular molecules associated with the residue. Resolving this possibility will require controls in which mechanically or thermally lysed, non-fermented CJLP243 cells (a “CJLP243 Lysate add” control) and, separately, a cell-free autolysate cleared of particulate debris are added to the unfermented substrate, together with direct compositional analysis of the PDIF. Because lysozyme does not cleave nucleic acids, DNase and RNase treatment of the PDIF, in the same format as the existing enzyme panel, would additionally provide a direct test of the adsorbed-nucleic-acid hypothesis.

### 3.7. Fermentation-Associated Adaptation of Pellet-Associated Amino Acid Pools

**Figure 8:**
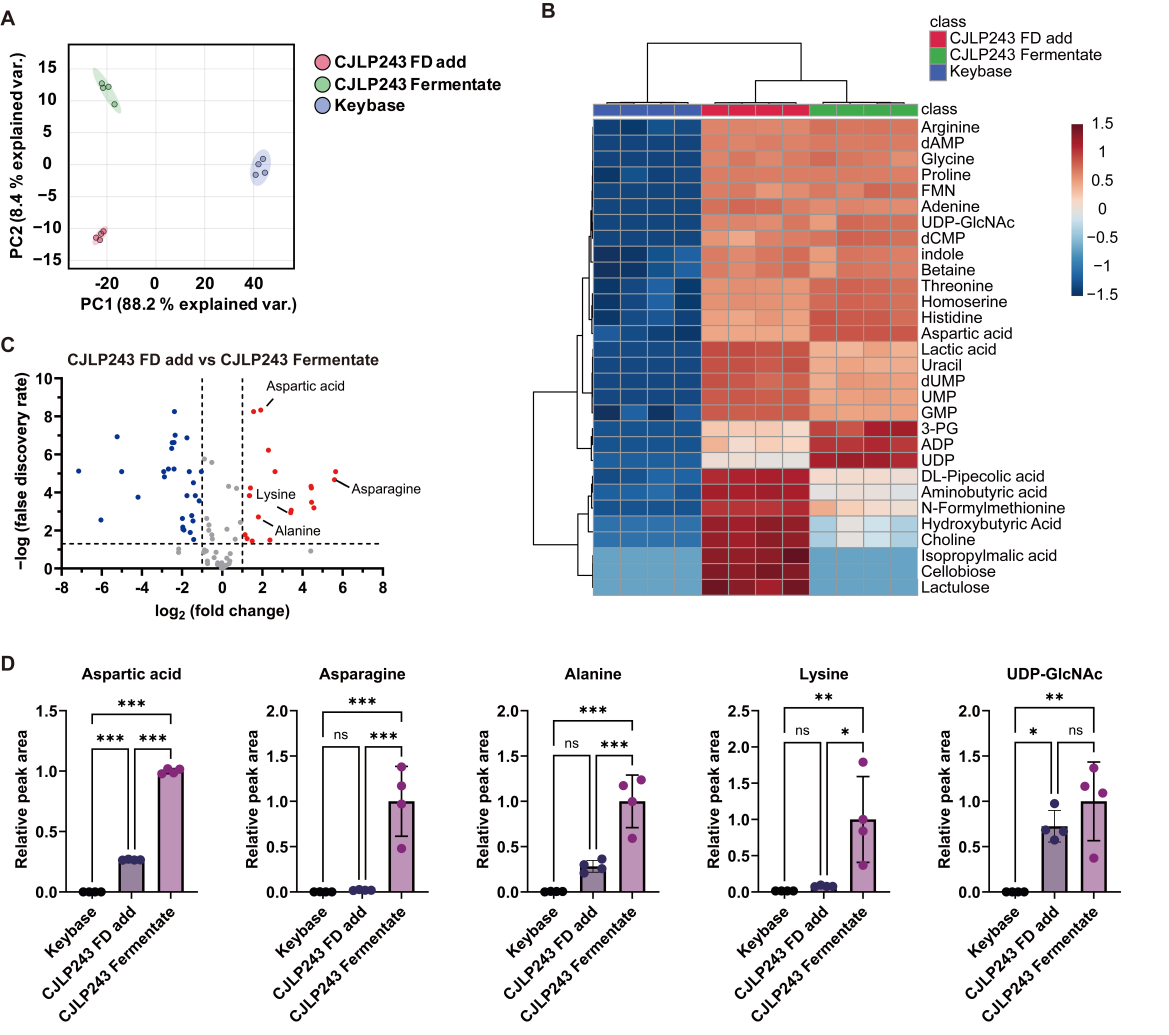
Targeted LC–MS/MS-based metabolomics reveals process-linked metabolic adaptation associated with CJLP243 fermentation. (**A**) Principal component analysis (PCA) of targeted metabolite profiles, showing group separation according to fermentation state. (**B**) Heatmap of significantly altered metabolites among Keybase, CJLP243 Fermentate, and CJLP243 FD add. (**C**) Volcano plot of metabolites altered in the CJLP243 Fermentate compared with the CJLP243 FD add. (**D**) Relative abundance of selected metabolites associated with fermentation-linked metabolic adaptation, including aspartic acid and other metabolites related to stem-peptide precursor metabolism. The CJLP243 Fermentate displayed a distinct metabolic profile compared with CJLP243 FD add, indicating that fermentation was accompanied by coordinated metabolic changes that paralleled acquisition of the immunostimulatory phenotype. For panel D, statistical significance was assessed by one-way ANOVA. Significance is denoted as * *p* < 0.05, ** *p* < 0.01, *** *p* < 0.001; ns, not significant.

To examine whether the acquisition of immunostimulatory activity was accompanied by metabolic changes, targeted LC–MS/MS analysis was performed on the cell pellets of Keybase, CJLP243 FD add, and CJLP243 Fermentate (Figure 8, Table S1). Heatmap and principal component analysis showed that CJLP243 Fermentate formed a metabolically distinct group relative to Keybase and CJLP243 FD add (Figure 8A,B). These results indicate that CJLP243 fermentation was accompanied by changes in the metabolite profile of the pellet-associated material. Experimentally, among the differentially altered metabolites (Figure 8C), aspartate showed the greatest enrichment relative to Keybase and was also significantly higher than in CJLP243 FD add. Alanine, asparagine, and lysine were likewise significantly increased, whereas UDP-N-acetylglucosamine, the precursor of the peptidoglycan glycan backbone, did not differ significantly from CJLP243 FD add (Figure 8D).

The PGN stem peptide of *L. plantarum* is L-Ala–D-Glu–*meso*-DAP–D-Ala, and *meso*-diaminopimelic acid (*meso*-DAP) is synthesized from aspartate-derived intermediates [30,31]. Alanine and glutamate can similarly supply, after racemization, the D-Ala and D-Glu stem-peptide residues assembled by Mur ligases [32]. Thus, the observed increase in alanine, together with the established role of glutamate in D-Glu synthesis, is consistent with an expanded supply of stem-peptide precursors under acidic fermentation conditions [11]. Targeted metabolomics reflects steady-state metabolite pools rather than direct PGN synthesis or turnover. The enrichment of aspartate and alanine therefore reports pool sizes rather than incorporation into the cell wall. The concomitant increase in lysine, a downstream product of the DAP pathway, is also consistent with altered DAP-associated metabolism during fermentation [31]. Notably, the enrichment of these amino acids without a comparable change in UDP-N-acetylglucosamine is compatible with a selective increases in stem-peptide-related amino acid pools rather than with coordinated upregulation of the entire PGN biosynthetic pathway. At the same time, free amino acid pools also reflect global nitrogen metabolism and acid-stress adaptation [11] and, in a plant-based matrix, may receive contributions from proteolysis of substrate-derived proteins; steady-state pool measurements alone cannot separate these contributions. The observed enrichment is therefore described as fermentation-linked metabolic adaptation compatible with an expanded precursor supply.

The cell wall-associated interpretation itself rests primarily on the fractionation and enzyme-sensitivity results. The major immunostimulatory activity of CJLP243 Fermentate was enriched in the PDIF and was markedly reduced by lysozyme treatment (Figure 2B,C and Figure 6B,C). *Meso*-DAP-containing peptidoglycan fragments are recognized by NOD1 in established biological systems [33,34]; in the present study, this relationship is considered only as a biochemical context for the PGN-associated interpretation and not as evidence that NOD1 mediates the observed cytokine response. Accordingly, the LC–MS/MS data should be interpreted as supportive rather than direct structural evidence, because *meso*-DAP-containing muropeptides were not directly quantified in this study. Taken together, the targeted metabolomics results suggest that CJLP243 fermentation induced metabolic adaptation, with aspartic acid enrichment providing a possible biochemical link to *meso*-DAP-related peptidoglycan metabolism. When considered together with the lysozyme-sensitive cytokine response, these findings are compatible with the working model that CJLP243 fermentation generates or exposes a cell wall-associated structure with macrophage-stimulatory activity.

### 3.8. Proposed Model for CJLP243 Fermentate-Mediated Macrophage Activation

**Figure 9:**
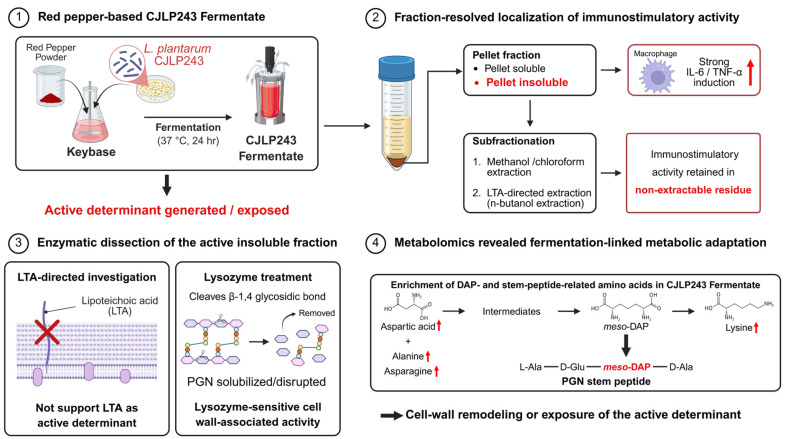
Proposed working model of fermentation-dependent cell-wall-associated immunostimulation in the red pepper-based CJLP243 Fermentate. CJLP243 fermentation of the red pepper-based substrate generates or exposes an immunostimulatory determinant that localizes to the PDIF and remains associated with non-extractable residual debris. LTA-directed analysis did not support lipoteichoic acid as the principal active component, whereas lysozyme sensitivity demonstrated structural dependency on a lysozyme-sensitive determinant within the PDIF, in which peptidoglycan-associated material is a plausible candidate. Targeted metabolomics revealed fermentation-linked metabolic adaptation of pellet-associated metabolites, including aspartic acid and selected amino acids related to peptidoglycan stem peptide precursor metabolism. These changes are shown as a proposed meso-DAP-related peptidoglycan precursor context that may accompany cell-wall remodeling or exposure of the active PDIF. This illustration was created using BioRender.com.

Taken together, the results support a working model in which red pepper-based fermentation with CJLP243 generates an insoluble, cell wall-associated fraction capable of inducing macrophage cytokine production (Figure 9). Consistent with the preceding sections, this activity was fermentation-dependent, was enriched and reproducibly recovered in the PDIF, was retained in the non-extractable residue after solvent fractionation, was strongly reduced by lysozyme, and was not attributable to LTA (Figure 1, Figure 2, Figure 3, Figure 4, Figure 5 and Figure 6). The main significance of this study is the stepwise localization and characterization of immunostimulatory activity within a fermented food matrix. Many studies on fermented foods evaluate biological activity using whole extracts, culture supernatants, or bacterial biomass. In contrast, the present study shows that the macrophage-stimulatory activity of the CJLP243 Fermentate was concentrated in an insoluble, fermentation-dependent, lysozyme-sensitive fraction. This finding distinguishes the CJLP243 Fermentate from systems in which functional activity is attributed mainly to soluble metabolites or general bacterial cell addition. It also suggests that fermentation-associated cell-wall remodeling or cell wall–matrix complex formation may be an important contributor to the functionality of lactic acid bacteria-fermented food materials.

These observations can be integrated into a mechanistic hypothesis for how fermentation generates a particulate active fraction. During fermentation, organic-acid production by CJLP243 would be expected to acidify the matrix. Such acidification would increase the protonation of anionic groups on the bacterial surface—including peptidoglycan carboxylates and phosphate groups in wall and lipoteichoic acids, with the charge of teichoic acids additionally modulated by D-alanylation [35]—and of galacturonic acid carboxyl groups in red pepper-derived pectic dietary fibers [36]. The resulting decrease in surface charge on both components would reduce electrostatic repulsion and, together with hydrophobic and hydrogen-bonding interactions, favor co-assembly of cell-wall fragments with insoluble plant fibers into a hybrid cell wall–matrix carrier, consistent with the fermentation-dependent changes in the surface properties of *lactobacilli* reported previously [10]. Because particulate, repetitively organized surfaces can be taken up and sensed by innate immune cells more efficiently than the corresponding soluble molecules [37], such a macro-carrier could enhance the multivalent presentation of putative peptidoglycan-associated motifs to macrophages, offering a plausible explanation for the strict partitioning of activity into the PDIF. Future studies incorporating measurements of zeta potential, particle size, and particle composition, together with assessments of receptor engagement, will be needed to directly test and further refine the proposed charge-dependent carrier model.

The findings are also relevant to postbiotic-oriented food development, but the terminology should be applied cautiously. According to the current consensus definition, postbiotics are preparations of inanimate microorganisms and/or their components that confer a health benefit on the host [3]. The CJLP243 Fermentate described here contains microbial structural components with in vitro immunostimulatory activity, but host-level benefit has not yet been demonstrated. Therefore, the material is best described at this stage as a fermentation-derived, cell wall-associated immunostimulatory fraction or a postbiotic candidate, rather than as a confirmed postbiotic product.

Overall, this study demonstrates that red pepper-based fermentation with CJLP243 reproducibly generates a PDIF with macrophage cytokine-inducing activity. The activity was not explained by soluble metabolites, simple addition of bacterial biomass, or LTA enrichment. Instead, the evidence supports the involvement of a fermentation-dependent, lysozyme-sensitive, cell wall-associated determinant, within which peptidoglycan-associated material remains a plausible but not exclusively demonstrated candidate. These findings provide a mechanistic basis for the development of CJLP243-fermented red pepper material as a process-defined functional food ingredient and offer a useful framework for evaluating cell wall-associated activity in other fermented food systems.

### 3.9. Study Limitations

J774A.1 macrophages served as a validated, well-characterized model for bioactivity-guided fractionation, allowing cytokine-inducing activity to be tracked across successive fractions. Several limitations of the present study should nonetheless be considered. First, although the enzyme-sensitivity profile indicates structural dependency on a lysozyme-sensitive cell-wall assembly, the precise muropeptide composition, degree of cross-linking, and higher-order architecture of the PDIF were not determined, and the active molecule(s) were not directly identified. Second, all biological readouts were obtained using a single immortalized murine macrophage cell line; cell-line-specific receptor repertoires and signaling thresholds may differ from those of primary macrophages and may not recapitulate responses in the intact host, so cytokine induction in this system does not establish a physiological immune benefit in vivo. Third, engagement of host pattern recognition receptors (PRRs) was not directly validated. Fourth, a contribution of intracellular material released by fermentation-associated autolysis and subsequently adsorbed onto the insoluble matrix cannot be formally excluded. Fifth, the targeted metabolomic data describe steady-state metabolite pools, which are influenced by substrate proteolysis and general stress adaptation as well as by cell-wall precursor supply, and therefore do not report on cell-wall architecture.

Addressing the remaining structural and translational questions will require mutanolysin digestion followed by UHPLC–MS/MS muropeptide mapping, meso-DAP quantification, and compositional (glycan and protein) analysis of the PDIF, together with reconstitution using purified peptidoglycan or defined muropeptides, to identify the active molecule(s); PRR reporter assays, such as HEK-Blue reporter cells for TLR2, NOD1, and NOD2, together with receptor-specific inhibition, to define the recognition pathway; and validation in primary macrophages (e.g., murine bone marrow-derived macrophages and human peripheral blood monocyte-derived macrophages), additional innate immune cell models, and appropriate in vivo models to establish physiological significance.

## 4. Conclusions

This study characterized the immunostimulatory property of a red pepper-based fermentate produced with *L. plantarum* CJLP243 and identified where that activity was localized within the fermented system. The cytokine-inducing effect was strongly dependent on fermentation and was not reproduced by simple addition of the strain to the substrate. Fractionation analyses further showed that the activity was concentrated in the PDIF rather than in supernatant-derived soluble fractions, indicating that the principal active determinant was associated with cell-linked structural material rather than diffusible metabolites alone. The reproducible detection of this activity across independent production batches further supports the view that the active PDIF represents a process-linked property of the CJLP243 fermentation system.

Structural analyses narrowed the likely basis of this activity. Solvent-based subfractionation retained the activity in the residual debris fraction, whereas LTA-directed extraction, MALDI-TOF MS, and phosphate analysis did not provide evidence that lipoteichoic acid was the major active component. In contrast, the marked loss of activity after lysozyme treatment is consistent with a lysozyme-sensitive, cell wall-associated determinant as a plausible source of the observed cytokine responses. These findings support an interpretation centered on a cell wall-associated insoluble structure generated or exposed during fermentation. Targeted LC–MS/MS-based metabolomics additionally showed that CJLP243 fermentation was accompanied by distinct metabolic adaptation, including enrichment of selected metabolites such as aspartic acid, which may serve as process-linked signatures associated with acquisition of the active state.

The present work goes beyond describing the bioactivity of a fermented material and instead defines how fermentation determines the localization and physicochemical nature of immunostimulatory activity in a red pepper-based food system. These findings provide a useful basis for the development of fermentation-derived functional ingredients and support future efforts to establish process-relevant markers for quality control, batch consistency, and mechanism-guided optimization of food fermentates with host-defense-related functionality.

## Data Availability

The original contributions presented in this study are included in the article/Supplementary Materials. Further inquiries can be directed to the corresponding authors.

## Supplementary Materials

The following supporting information can be downloaded at https://www.mdpi.com/article/10.3390/foods15152736/s1, Table S1: The targeted metabolomic analysis dataset.

## Abbreviations

The following abbreviations are used in this manuscript:

ANOVA	Analysis of variance
DHB	2,5-Dihydroxybenzoic acid
DMEM	Dulbecco’s modified Eagle’s medium
DMSO	Dimethyl sulfoxide
ELISA	Enzyme-linked immunosorbent assay
FD	Freeze-dried
FDR	False discovery rate
HF	Hydrofluoric acid
HIC	Hydrophobic interaction chromatography
HPLC	High-performance liquid chromatography
IL-6	Interleukin-6
LC–MS/MS	Liquid chromatography–tandem mass spectrometry
LPS	Lipopolysaccharide
LTA	Lipoteichoic acid
MALDI–TOF MS	Matrix-assisted laser desorption/ionization time-of-flight mass spectrometry
meso-DAP	meso-Diaminopimelic acid
MRM	Multiple reaction monitoring
MS	Mass spectrometry
NOD1	Nucleotide-binding oligomerization domain-containing protein 1
NOD2	Nucleotide-binding oligomerization domain-containing protein 2
PBS	Phosphate-buffered saline
PCA	Principal component analysis
PDIF	Pellet-derived lysate-insoluble fraction
PGN	Peptidoglycan
PRR	Pattern recognition receptor
SD	Standard deviation
TLR2	Toll-like receptor 2
TMB	3,3′,5,5′-Tetramethylbenzidine
TNF-α	Tumor necrosis factor-α
UDP-GlcNAc	Uridine diphosphate N-acetylglucosamine
UV–vis	Ultraviolet–visible
